# Phylogenetic analysis of museum specimens of houting *Coregonus oxyrinchus* shows the need for a revision of its extinct status

**DOI:** 10.1186/s12862-023-02161-7

**Published:** 2023-09-27

**Authors:** R. Kroes, Y. Winkel, J. A. J. Breeuwer, E. E. van Loon, S. P. Loader, J. S. Maclaine, P. F. M. Verdonschot, H. G. van der Geest

**Affiliations:** 1 Department of Freshwater and Marine Ecology, Institute for Biodiversity and Ecosystem Dynamics, University of Amsterdam, P.O. Box 94240, 1090 GE Amsterdam, The Netherlands; 2 Natural History Museum, Cromwell Rd, South Kensington, London, SW7 5BD UK; 3 Wageningen Environmental Research, Wageningen University & Research, Droevendaalsesteeg 3-3 A, Wageningen, 6708 PB The Netherlands

**Keywords:** Phylogeny, Coregonids, Houting, Ancient DNA, IUCN, Linnaeus, Nature conservation

## Abstract

According to the IUCN Red List the anadromous houting *Coregonus oxyrinchus* is categorized as ‘extinct’. However, this extinct status might be incorrect because taxonomic difference between *C. oxyrinchus* and the closely related C*. lavaretus* is based on a disputable morphological comparison. Also, phylogenetic studies on mtDNA only focused on recent obtained coregonids. We are the first to perform a mtDNA analysis on both historic and recent specimens, including the syntype specimen which was used for species description by Linnaeus originally. Two primer pairs for mitochondrial *CytB* and *ND3* were used to extract sequences for phylogenetic analysis. Sequences from 14 out of 21 *C. oxyrinchus* museum specimens were successfully obtained and compared with sequences from recent obtained *C. lavaretus*. The sequences were combined with GenBank data from a previous phylogenetic study on houting to create a phylogenetic tree and two minimum spanning haplotype networks. Results show that *C. oxyrinchus* and *C. lavaretus* form a clade with limited genetic variation. Low bootstrap values also show weak support for geographical patterns in distribution of mitochondrial haplotypes. Statistical analysis of the haplotype networks also shows that historic and recent specimens are similar species. Our results suggest that *C. oxyrinchus* is a junior synonym of *C. lavaretus*. A definitive taxonomic revision could not be made because only *CytB* sequencing was successful for the syntype specimen. We discuss taxonomic consequences and the species-specific focus in nature conservation. We propose a shift in nature conservation to a more functional approach based on traits rather than species.

**Supplementary Information:**

The online version contains supplementary material available at 10.1186/s12862-023-02161-7.

## Background

Containing nearly 80 species the whitefish genus *Coregonus* is a diverse salmonid group inhabiting lakes, rivers and open fresh and sea water habitats [1]. The genus is also of high conservation interest given the high number of extinct and endangered species. However, the complex evolution of phenotypic traits has caused taxonomic confusion resulting in a number of nomenclatural issues [2–4].

One of the representatives is the anadromous houting or ‘North Sea houting’ *Coregonus oxyrinchus*, once geographically widespread in the Wadden Sea area and the coastal zones of the southern North Sea [5]. This species was first described by the Dutch ichthyologist Gronovius in 1754–56, although not in binominal nomenclature and with genus ‘*Salmo’* rather than ‘*Coregonus’* [6]. Linnaeus used the work of Gronovius to describe the species in the 10^th^ and 12^th^ edition of Systema naturae. Dried fish skins from Gronovius’ collection used for the species description were later incorporated in the collection of the Natural History Museum of London, including the type specimen for *C. oxyrinchus* [6].

Present-day, according to the IUCN Red List *C. oxyrinchus* is considered extinct [7]. This claim is based on a comparison of two morphological traits, snout length and number of gill rakers, taken from museum specimens (non-type material) and recent collected coregonids by Freyhof & Schoter [8]. However, phylogenetic studies from the last two decades challenge the taxonomic status of *C. oxyrinchus* and its IUCN listing as being extinct [5, 9–15]. Morphological traits might be plastic and change during the lifetime of a fish, for example when they migrate back and forth between fresh and salt water or change diet during growth. Also, homoplasy of morphological traits is common in coregonids because of rapid adaptation and differentiation to trophic ecology, reproductive behavior and diet acquisition [16–19]. This homoplasy hypothesis is supported by the absence of a relationship between the mitochondrial DNA (mtDNA) phylogeny and the number of gill-rakers within the *C. lavaretus* complex [15, 20]. Furthermore, mitochondrial genome sequences of European *Coregonus* spp. are less than 2% different (313 out of 16,600 nucleotides) suggesting that genetic diversification is recent and perhaps the result of isolation by distance [20]. Besides questionable morphologic traits, also geographic origin seem to be used for identification of *C. oxyrinchus*. Specimens originating from the Wadden Sea area and the coastal zones of the southern North Sea are usually identified as *C. oxyrinchus*. Specimens from the Baltic and its tributary rivers are usually identified as *C. lavaretus* [20, 21].

Because of the morphological and phylogenetic discussions, it remains unclear if *C. oxyrinchus* is a distinct species or an ecotype of *C. lavaretus* to date [22]. Until detailed comparisons are made between type, historic and current material it cannot be ruled out that the *C. oxyrinchus* is a separate lineage. Therefore, in this study our aim is to examine molecular phylogenetic patterns of European whitefish to better understand the groups taxonomy. Specifically, we will compare various parts of older and recent *Coregonus* genomes, including never previously sampled historic material, to reveal the taxonomic status of *C. oxyrinchus*. We hypothesize that ancient and recent *Coregonus* spp. form a clade, and that this will have taxonomic and conservation implications for the species *C. oxyrinchus*.

## Results

### Phylogenetic analysis

Thirty-eight successful PCR samples were sent for sequencing of which *n* = 23 sequences remained with consistent peak patterning of which *n* = 9 recent obtained and *n* = 14 museum specimens (see S.1 for sequence success overview and GenBank accession numbers). Together with the dataset from Østbye et al. [15], the resulting dataset comprised 8 specimens with 187 aligned bp for *CytB* and 187 bp for *ND3*. The percentage of phylogenetically informative (PI) sites in the aligned dataset was 4.5% for *CytB* and 7.8% for *ND3* (Table 1).

**Table 1:** General information on alignment of the markers and substitution models used for creating Maximum Likelihood trees. #sequences gives the number of sequences available per marker in this study, whereas the ones in brackets are from Østbye et al*.* [15]. Positions is the number of nucleotides per alignment, V stands for the number of variable sites in each alignment, Pi is the number of phylogenetically informative sites per alignment, %Pi explains the percentage of the sequence that is phylogenetically informative. Model is the substitution model test (log likelihood-ratio test) carried out in MEGA7 to find the best model for constructing a Maximum Likelihood (ML) tree and base composition explains the nucleotide composition of the alignment

	**Genetic marker**	#sequences	Positions	V	Pi	%Pi	Model	Base composition			
								T	C	A	G
Mitochondrial	Cytochrome b (*CytB*) & NADH dehydrogenase 3 (*ND3*)	22 + (62)	460	51	27	5.9	K2 + G	0.301	0.342	0.199	0.158

**Fig. 1:**
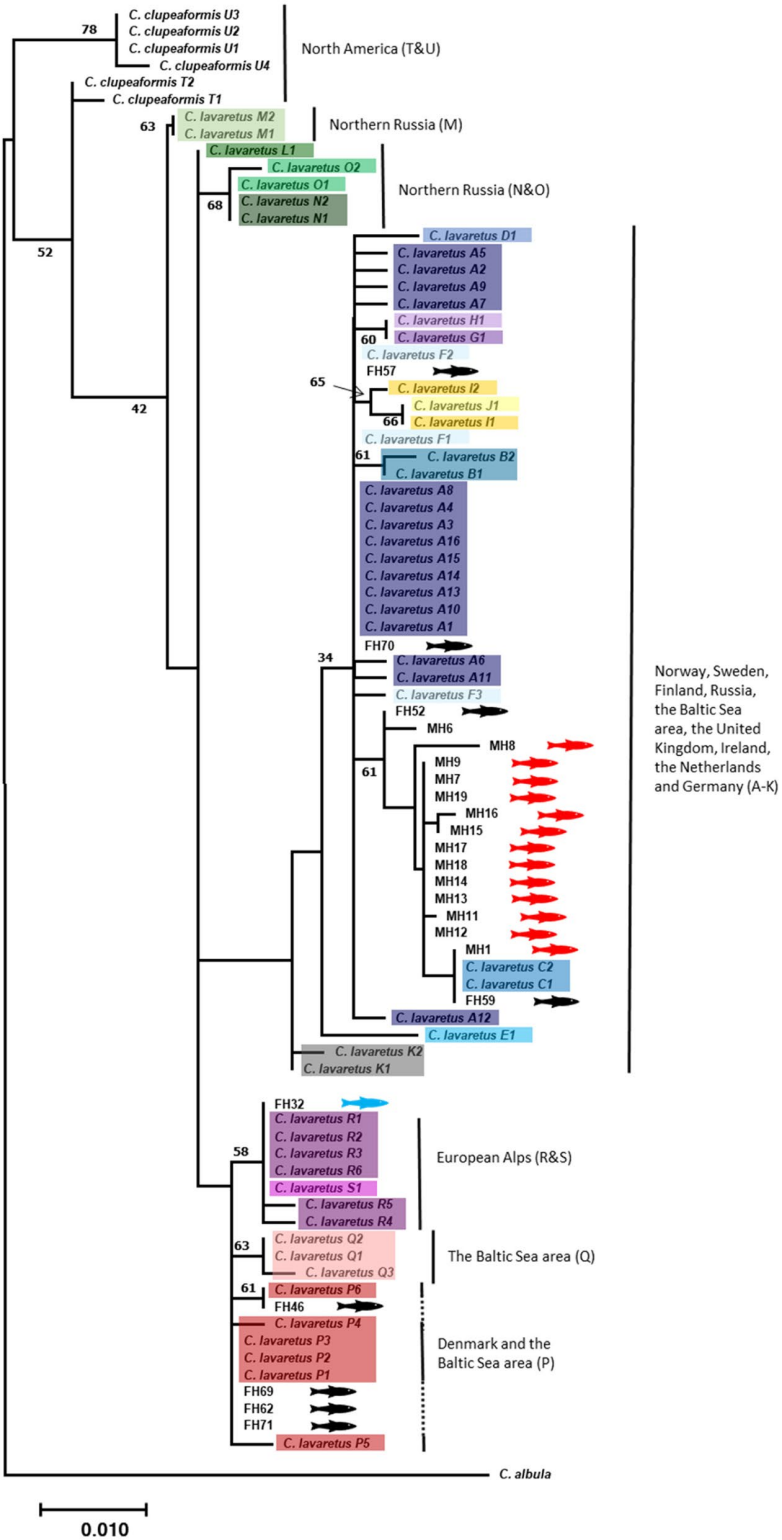
The Maximum Likelihood phylogenetic tree of the mtDNA (*CytB* + *ND3*) sequences of *Coregonus* spp. with node support from 500 bootstraps on branches. The geographical distribution of major branches is given. The 1-step nested clades are marked in colour. Representatives from *Coregonus clupeaformis* and *Coregonus albula* are included as the genetic outgroup. The recent obtained specimens of *Coregonus* spp. are indicated by FH and a black fish behind the name for the ones from the Netherlands and a blue fish for the one from the fish farm in Germany. Museum specimens of *Coregonus* spp. are indicated by MH with a red fish. The clades within the geographical distribution are given in brackets. The tree was drawn to scale, with branch lengths measured in the number of substitutions per site

This dataset was used to construct a phylogeny using the maximum likelihood method and Kimura 2-parameter substitution model with a discrete Gamma distribution (5 categories (+ G, parameter = 0.1463)) to generate bootstrap support for clades. The topology of this tree resembled the phylogeny of Østbye et al. [15] and the same clades appeared in our extended phylogeny (Fig. 1). Only small differences in the splitting order of some clades were observed. These were probably due to the small number of informative sites and inconsistencies between informative sites. The latter is evidenced by the low bootstrap values, less than 70%, for these clades. In addition, most of the informative sites separated *C. lavaretus* from *C. clupeaformis* and *C. albula*, which were used as outgroups.

The museum specimens, collected at the turn of the twentieth century from Ireland, the United Kingdom, the Netherlands, and Belgium, clustered together with recent specimens from Norway, Sweden, Finland, Russia and the Baltic Sea area [15] and two recent obtained specimens in this study from freshwater bodies in the Netherlands. However, bootstrap support for this clade was low (= 62%).

All museum specimens belonged to a larger weakly supported group of specimens indicated by Østbye et al. [15] that have been collected from the Barents Sea (part of the Arctic Ocean), the Baltic Sea and its Scandinavian freshwater tributaries, and Danish tributaries to the North Sea (clade A-K). Sequence differences between specimens in this group were very small (d = 1.51 bp, 0.4% sequence divergence) and did not exceed more than 5 base pair differences, all in *ND3*, indicating that the museum specimens are not different from recent obtained specimens. Sequencing of the syntype and neotype specimens was only successful for *CytB* and were therefore not included in the concatenated phylogeny of *CytB* and *ND3*. Recent obtained specimens from the Netherlands were spread out across various other clades (P, R, C, A and F) that were indicated by Østbye et al. [15]. The recent obtained specimens in the Netherlands and Germany do not form a monophyletic clade nor do they belong to a specific clade and therefore are not a distinctive species.

**Fig. 2:**
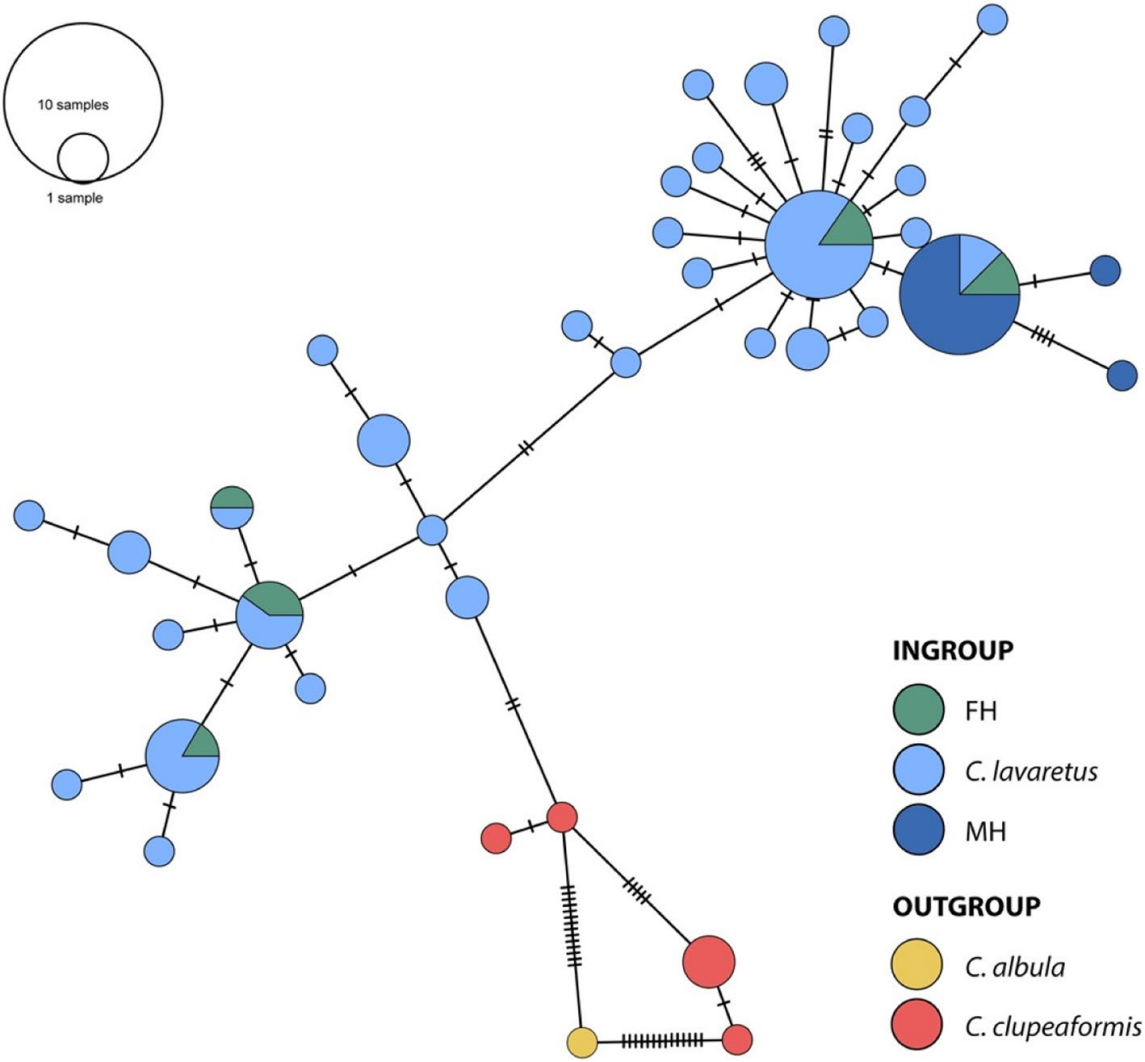
Minimum spanning haplotype network of the mtDNA (*CytB* + *ND3*) sequences of *Coregonus* spp. The relative contribution of the different taxonomic groups per node are marked in colour. Representatives from *C. clupeaformis* and *C. albula* are selected as the genetic outgroups. Dashes indicate number of substitutions; the length of the edges is not informative

**Fig. 3:**
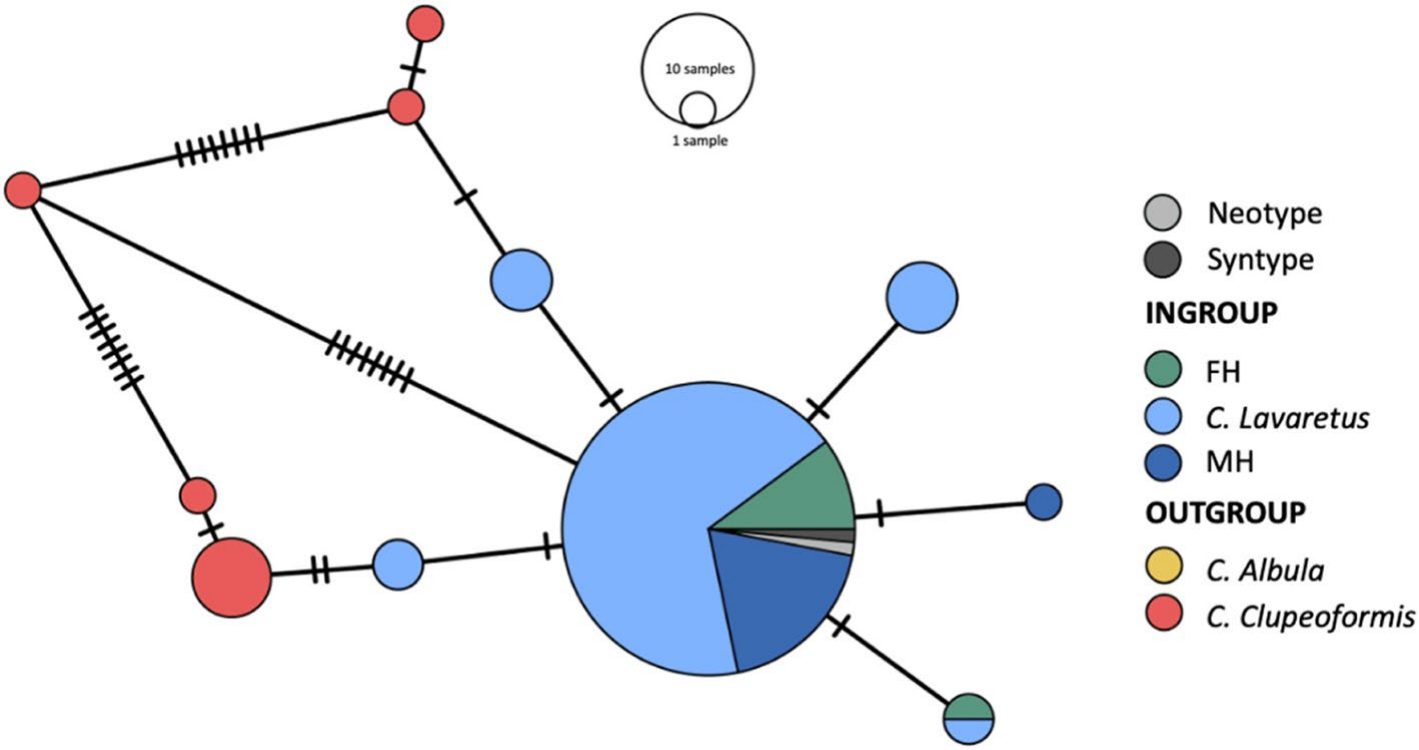
Minimum spanning haplotype network of the mtDNA (*CytB*) sequences of *Coregonus* spp. The relative contribution of the different taxonomic groups per node are marked in colour. Representatives from *C. clupeaformis* and *C. albula* are selected as the genetic outgroups. The neotype and syntype were added to this network. Dashes indicate number of substitutions; the length of the edges is not informative

The minimum spanning haplotype network for both *CytB* and *ND3* supports the above results as specimens from the ingroup (*C. lavaretus*, museum and recent obtained specimens) cluster together and are separated from the outgroup (*C. albula* and *C. clupeaformis)* (Fig. 2). P-distance within groups was 0.01 (ingroup) and 0.02 (outgroup). Between ingroup and outgroup, p-distance was 0.03. AMOVA shows that variation between groups was significant (AMOVA: Fixation index Phi_ST = 0.818, Phi_SC = 0.517, Phi_CT = 0.622; Significance of Phi_ST, Phi_SC and Phi_CT < 0.001). In the minimum spanning haplotype network for only *CytB*, the neotype and syntype were additionally added to the ingroup. The *CytB* network shows that both the syntype and the neotype specimens cluster with the other included *C. lavaretus* and *C. oxyrinchus* specimens from the ingroup (Fig. 3). P-distance within groups was 0.01 (ingroup) and 0.02 (outgroup). Between ingroup and outgroup, p-distance was 0.02. AMOVA shows that variation between groups was not significant (AMOVA: Fixation index Phi_ST = 0.818, Phi_SC = 0.517, Phi_CT = 0.622; Significance of Phi_ST < 0.001, Phi_SC = 0.063 and Phi_CT = 0.09). However, *CytB* sequences from both type specimens were equal to both museum and recent obtained specimens in the clades C and E from the phylogenetic tree and differed 1 basepair from recent obtained specimens in clades R and P. Thus, based on this sequence divergence between ingroup and outgroup there is no strong genetic signal to further divide the studied European *Coregonus* specimens in different (phylogenetic) species.

## Discussion

### Phylogenetics

In 2008, *C. oxyrinchus* was categorized as ‘extinct’ on the IUCN Red List of Species. This claim is based on the morphological analysis of Freyhof and Schöter [8] on gill raker numbers. We sampled both specimens they used from the Natural History Museum in London (MH17 and MH20, original identification BMNH1844.11.11.15 and BMNH1862.11.20.1) as well as the syntype (MH21, original identification BMNH 1853.11.12.160). MH17 was included in our phylogenetic tree and the *CytB* + *ND3* haplotype network. The syntype and neotype were also included in the *CytB* haplotype network. Our mtDNA sequence analysis shows that all older and recent *Coregonus* spp., formerly classified as *C. oxyrinchus* and *C. lavaretus* respectively, clustered together and did not form separate clades or lineages in the phylogenetic tree. This conclusion is supported by the low bootstrap values on branches. Also, the older *C. oxyrinchus* show no monophyletic geographic distribution in comparison to the recent obtained *C. lavaretus*. Statistical analysis on the *CytB* and *ND3* haplotype network further supports our conclusion. The mean p-distance within ingroup and outgroup was 2–3 times lower than p-distance between the groups. Variance between ingroup and outgroup for *CytB* and *ND3* was also significant as shown by AMOVA. Observed significant variance between determined species is caused by the diverse outgroups used in this study. Excluding the outgroup showed that there is no significant variance between taxonomic groups from the ingroup (significance of Phy_ST = 0,481). In the *CytB* haplotype network, the syntype and neotype also cluster with other taxonomic groups from the ingroup. Although no significant variance was found between ingroup and outgroup for *CytB*, this can be explained by the limited numbers of mutations between taxonomic groups and therefore less informative site of *CytB*. Thus, our hypothesis that ancient and recent *Coregonus* spp. form a single clade is accepted. This result has taxonomic and conservation implications towards recognition of the taxon *C. oxyrinchus*.

We argue for a revision of the IUCN conservation status ‘extinct’ of *C. oxyrinchus*. The currently used phenotypic traits weakly support the species status of *C. oxyrinchus*. Although not analyzed in the present study because of destructiveness of museum material, there is ample evidence from literature that gill raker numbers show high intraspecific variation, mainly because of rapid and recent evolution [12, 15, 17, 23]. This conclusion is supported by Etheridge et al. (2012) who showed that gill raker number is an inappropriate trait for taxonomic subdivision in UK-specific coregonids [24].

Previous phylogenetic studies on the *Coregonus* genus showed the presence of multiple distinctive lineages, mainly geographically clustered [5, 15]. However, bootstrap values are low to moderate and only weakly supported the geographic clusters indicated by Østbye et al. [15]. In addition, Hansen et al. [5] only used a small geographic area for their samples. Identification of geographical clusters of *Coregonus* spp. is further confused by introgression and admixture which are attributed to both natural and anthropogenic events, such as restocking [9, 20, 25–27]. More detailed studies with microsatellites more strongly supported the distinction of different *Coregonus* populations [16, 26]. A complete mtDNA genome analysis of Jacobsen et al. [20] showed that the anadromous “North Sea houting” from the Vidå river differs from non-anadromous or freshwater inhabiting coregonids. However, these morphological, geographical and genetic criteria are used to discriminate within species level, not between.

Based on plasticity of morphological traits used for identification, weak support on geographically patterns in distribution of mitochondrial haplotypes and our analysis of older material, we conclude that North Sea Houting *C. oxyrinchus* is not and has not been a biological species. Our results suggest that *C. oxyrinchus* is a junior synonym of *C. lavaretus*. A taxonomic revision awaits further analysis of the syntype from the Gronovius collection since alternative relatively shorter markers should also be sequenced to obtain sufficient phylogenetically informative sites. However, complications for further analysis are caused by limited amplicon length that can be obtained from older museum specimens. More importantly, morphological identification of specimens that are used for sequencing is uncertain.

Based on our findings we hypothesize that syntype and neotype will cluster together in future analyses as well. Since the syntype is extant in the Natural History Museum, the *C. oxyrinchus* neotype BMNH1862.11.20.1 designated by Freyhof and Schöter [8] is probably invalid. The absence of gill rakers for identification of the syntype specimen should not have been used as an argument to designate a neotype. This morphological trait is plastic and more important, the original type material for the first binominal species description from Linnaeus can be used for phylogenetic analysis.

### Management implications

In 2000, the European Union launched the Water Framework Directive (WFD) to restore the ecological status of European freshwater ecosystems. One of the measures to reach this goal was to reconnect water systems and hence mitigate migratory fish species [28]. Effects of these measures are often monitored by taxonomic inventories [29]. The IUCN uses these inventories as a primary source of distribution data. Because of the difficulties of fish taxonomy in general and coregonids in particular, legal problems consequently arise for taking the right actions to protect migrating fish if taxonomy fails. For example, in Europe houting *C. oxyrinchus* is both extinct (IUCN) and a present protected species (Bern convention, Habitats Directive, OSPAR). Without taxonomic agreement, it is unknown to which species the alive and swimming fish in Europe belong.

For ecosystem functioning, taxonomic inventories are less important. Recent studies show that ecosystem functioning and stability depends on functional diversity and its underlying genetics rather than taxonomic diversity [30–32]. Villéger et al. [32] also show that anthropogenic impacts have a six-fold higher effect on functional homogenization than taxonomic homogenization, with stronger homogenization when fish species are translocated. The latter happened with coregonids that originated from a Danish population and were reintroduced to the Rhine area in the 1990’s [33]. A study from Borcherding et al. [9] showed facultative migration of *Coregonus* spp. (classified as *C. oxyrinchus* in their study) in Lake IJsselmeer, but populations in other lakes and rivers in The Rhine delta have not been studied yet. It is therefore unknown to what extend the reintroduced fish express their original anadromous way of life and how they affect ecosystem functioning.

## Conclusions

Functional and phylogenetic diversity in the genus *Coregonus* needs protection. Anadromous populations in the North Sea basin suffered the most from habitat fragmentation and degradation and almost disappeared in the 1980’s. A habitat restoration program probably saved the Danish North Sea population from extinction [21]. For taxonomic revision, the syntype from the Gronovian collection requires more detailed research. For the genus *Coregonus*, we propose that policies avoid the species specific focus in nature conservation. Instead management actions should focus on the restoration and functioning of water bodies, including restoring connectivity as stated in the WFD. To do so, more knowledge on the niche conditions of newly established coregonid populations is needed to fully understand its ecological role and conservation status.

## Methods

### Specimen samples

**Fig. 4:**
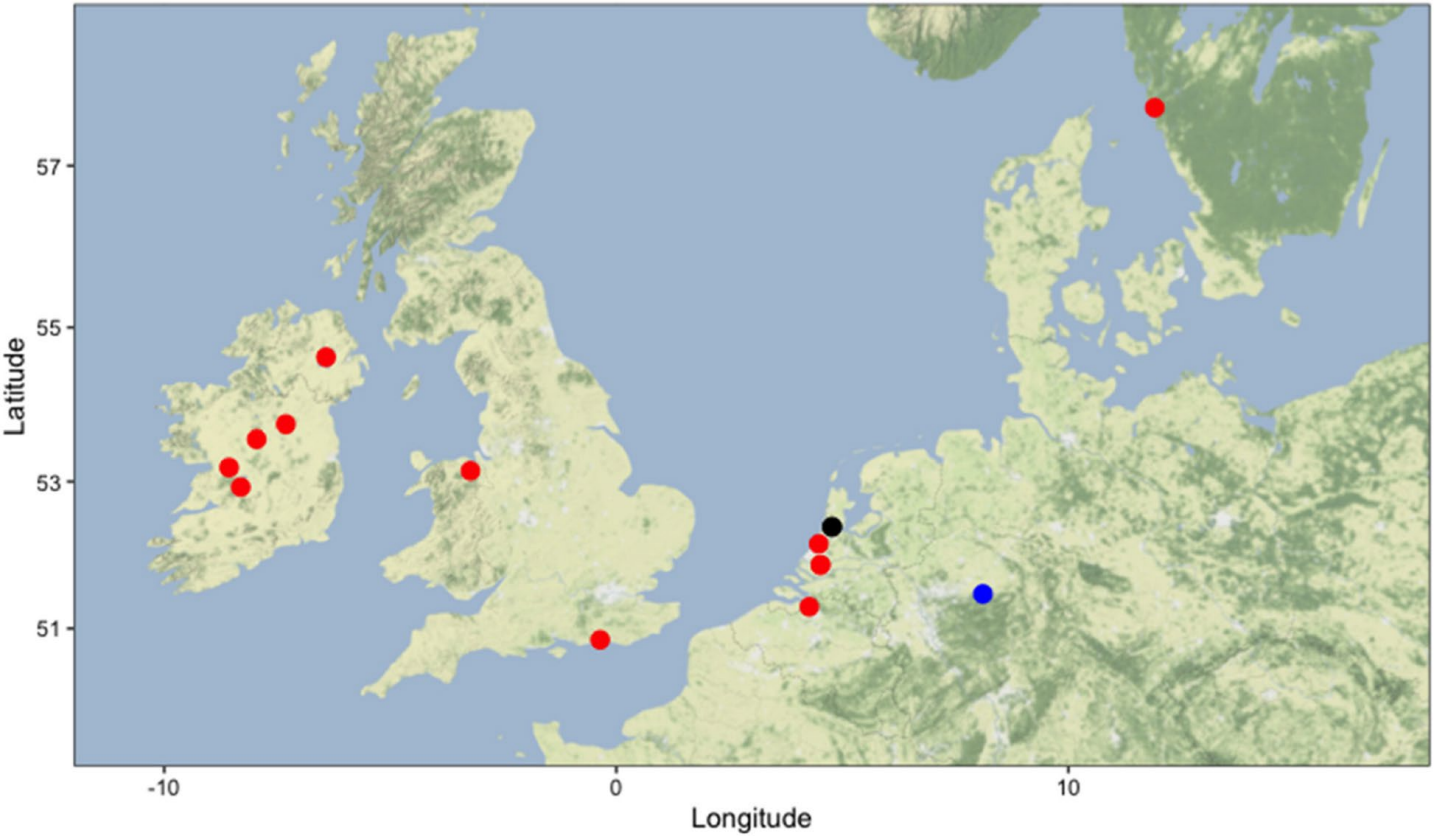
Origin of *Coregonus oxyrinchus* from the Natural History museum in London and recent obtained whitefish. The red dots indicate the origin of the museum specimens (for two specimens, the location was unknown, and therefore not plotted on the map). The black dot indicates the origin of the recent obtained specimens in the Netherlands. The blue dot indicates the origin of the specimens from the fish farm in Germany (Background source: stamen maps)

Pectoral fins from 20 specimens stored in ethanol and muscle tissue from the dried skin of one specimen of *Coregonus* spp. were sampled from the collection of the Natural History Museum in London. Museum specimens dated from 1844 to 1927 and were classified as *C. oxyrinchus*. The geographical origin of the specimens covered the sea, lakes and rivers in the United Kingdom, Belgium, the Netherlands, Sweden and two unknown origins. From the total of 21 sampled specimens, 19 were non-type material. The neotype designated by Freyhof and Schöter [8] (BMNH 1862.11.20.1) and the syntype of *C. oxyrinchus* (BMNH 1853.11.12.160, dried skin of *Salmo oxyrinchus* Linnaeus, 1758, former name of *C. oxyrinchus*) from the Gronovian collection were also sampled (Fig. 4 for geographic distribution and Supplementary S.1 for all sample data).

In addition, pectoral or tail fins from 23 specimens of *Coregonus lavaretus* from the collection of the Institute of Biodiversity and Ecosystem Dynamics were sampled. These specimens were offered to the Institute between 2014 to 2017 and originated from the Westeinderplassen and the Noordzeekanaal in the Netherlands were they were found dead. All specimens were stored at -80 °C.

Finally, 17 larvae from a *C. lavaretus* hatchery of the Ruhrverband in the Mohnesee (Germany) were obtained in 2017 (exact location included in S1). The tissue used in DNA analysis consisted of the entire individual stored in ethanol.

### DNA extraction

Tissue was cut into small pieces using sterilized scissors and dried on filter paper. Next, tissue was placed in 0.97 mL lysis buffer (200 mM Tris–HCl (pH 8.0), 100 mM EDTA and 250 mM NaCl), 5 μL Proteinase K (20 mg/mL) and 60 μL 10% SDS and incubated for a minimum of 2 h at 48°C. After incubation, DNA was extracted with phenol:chloroform:isoamyl alcohol and isopropanol precipitated with ammonium acetate [34]. The DNA pellet was dissolved in 50 μL water. In addition, 1 μL RNAse (10 mg/mL) was added to the DNA samples of the recent obtained specimens to digest RNA according to Younas [35]. Isolated DNA was stored at -20°C until further use.

To prevent cross-contamination between historic and recent obtained DNA samples, we extracted DNA from the museum samples (likely to have low DNA concentration) before the recent obtained samples. In addition, all extractions were carried out in a fume hood that was decontaminated by UV light for a minimum of 30 min before starting the actual extraction. After every extraction the pipettes were cleaned using 96% ethanol and during the extraction only filter pipet tips were used to avoid transfer of aerosols.

### PCR and sequencing

Two primer pairs for mitochondrial *CytB* and *ND3* previously used in phylogeographic research on European *Coregonus* species were used in this study [15, 36] (Table 2). Each PCR was done in a volume of 10 μL, containing 3.5 μL sterile milliQ water, 2 μL Phire hotstart Buffer (5x), 2 μL dNTP’s (1mM), 0.2 μL of each primer (10 μM), 0.1 μL Phire hotstart polymerase (Thermofisher) and 2 μL of template DNA (20–50 ng). As with DNA extraction, we also used filter pipet tips for preparing PCR’s. Each PCR reaction contained one negative and one positive sample. For the negative sample sterile milliQ water was used and for the positive sample a good DNA sample from a recent obtained specimen was used. PCR was performed 3 times until negatives were blank. PCR conditions for the mitochondrial primers were 30s at 98°C; 35 cycles of 10s at 98°C, 10s at 58°C and 20s at 72°C; 1 min at 72°C and hold at 10°C. After PCR, products were run on 1.5% agarose gel and checked for DNA quality, *i.e.* brightness and length of amplicon and presence of unspecific amplicons. Subsequently, 1 μL of each amplicons was Sanger sequenced by Macrogen Europe Laboratories in a final volume of 10 μL, 1 μL of the forward primer (10 μM) and 8 μL sterile milliQ water.

**Table 2:** *ND3* and *CytB* markers used for *Coregonus oxyrinchus*. The name stands for the part of the amplified gene. The forward and reverse primer give the sequence of the primer, length is the length of the sequence that this primer amplifies. Reference stands for the study where the primer is derived from

	Name	Forward primer (5’- > 3’)	Reverse primer (5’- > 3’)	Length	**Reference**
Mitochondrial	Cytochrome b (*CytB*)	CTTCGCCTACGCAATCCTAC	GGCTCATTCGAGGGCTTTAT	282 bp	Østbye et al. 2005 [15]
	NADH dehydrogenase 3 (*ND3*)	CATCACCATCGCACTATCCA	CCTCCTTGGGTTCACTCGTA	241 bp	Østbye et al. 2005, Schulz et al*.* 2006 [15, 36]

### Phylogenetic analysis

Low quality ends of sequences were trimmed using standard settings CLC Main Workbench 20.0.4 (Qiagen). After trimming, sequences were aligned per marker and observed differences were checked in the chromatograms by eye once to confirm that differences were true. Specimens with both *CytB* and *ND3* sequences available were concatenated by 100 N’s into a single sequence. The concatenated sequences were combined with the GenBank PopSet 1015647666 data from Østbye et al. [15].

To find the best nucleotide substitution model for each alignment a model test was implemented in MEGA version 7.0.26 [37]. The evolutionary history was inferred by using the Maximum Likelihood method based and the corresponding substitution model ss determined by Modeltest in MEGA. Initial trees for the heuristic search were obtained automatically by applying Neighbor-Join and BioNJ algorithms to a matrix of pairwise distances using the Maximum Composite Likelihood (MCL). The tree was drawn to scale, with branch lengths measured in the number of substitutions per site. Codon positions included were 1st + 2nd + 3rd + Noncoding. All positions containing gaps and missing data were eliminated. The dataset was bootstrapped 500 times to assess support for clades in the phylogeny with the highest log likelihood.

In addition to the phylogenetic tree, the same dataset was used to construct a minimum spanning haplotype network for *CytB* + *ND3* using PopArt [38]. In the network 5 groups were labeled: Crête-lafrenière et al. (2012) was used to select *C. albula* and *C. clupeaformis* for the outgroup, the ingroup comprised *C. lavaretus*, museum and recent obtained specimens [39]. An additional minimum spanning haplotype network was constructed for *CytB* only, in which the syntype and neotype were additionally added to the ingroup. The networks were used to analyze whether the outgroups from both networks were significantly different from the ingroups. For this, MEGA version 7.0.26 [37] was used to determine p-distance within and between groups. In addition, Phy-statistics were calculated within and between groups with AMOVA as implemented in PopArt [38].

### Supplementary Information

**Additional file 1: S.1. **Sample origin and analysis results. For each sample, code used in this study, original reference code, type status, GenBank accession numbers, species characteristics, origin and analysis results are given, if available. 'NA' refers to data that is not available.

**Additional file 2: S.2.** NCBI data for ND3. Given are sample data and ND3 analysis results for samples included in the phylogenetic tree and haplotype networks, as submitted to GenBank. See S.1 for accession numbers for corresponding samples.

**Additional file 3: S.3.** NCBI data for ND3. Given are sample data and ND3 analysis results for samples included in the phylogenetic tree and haplotype networks, as submitted to GenBank. See S.1 for accession numbers for corresponding samples.

## Data Availability

The datasets generated and/or analyzed during the current study are deposited under embargo at GenBank under accession numbers OP723928—OP723955 for *ND3* and OP723956—OP723986 for *CytB*. Accession numbers per sample are listed in table S.1 (supplementary data).
